# A Comprehensive Antioxidant and Nutritional Profiling of Brassicaceae Microgreens

**DOI:** 10.3390/antiox14020191

**Published:** 2025-02-07

**Authors:** Anja Vučetić, Olja Šovljanski, Lato Pezo, Nevenka Gligorijević, Saša Kostić, Jelena Vulić, Jasna Čanadanović-Brunet

**Affiliations:** 1 Faculty of Technology Novi Sad, University of Novi Sad, 21000 Novi Sad, Serbia; anja.saveljic@uns.ac.rs (A.V.); jvulic@uns.ac.rs (J.V.); 2 Institute of General and Physical Chemistry Belgrade, 11000 Belgrade, Serbia; latopezo@yahoo.co.uk; 3 Department of Experimental Oncology, Institute for Oncology and Radiology of Serbia, 11000 Belgrade, Serbia; nevenka.malesevic@ncrc.ac.rs; 4 Institute of Lowland Forestry and Environment Novi Sad, 21000 Novi Sad, Serbia; sasa.kostic@uns.ac.rs

**Keywords:** microgreens, Sango radish, kale, antioxidant activities, headspace analysis, pharmaceutical activities, HPLC analysis, Brassicaceae

## Abstract

Microgreens are gaining prominence as nutrient-dense foods with health-promoting activities while aligning with smart agriculture and functional food trends. They are rich in numerous bioactive compounds like phenolics, ascorbic acid, and carotenoids, which act as antioxidants, while also causing multiple other biological activities. Using advanced statistical methods, this study investigated Brassicaceae microgreens, identifying kale and Sango radish as standout varieties. Both contained 16 amino acids, with potassium and calcium as dominant minerals. Sugar and protein contents ranged from 4.29 to 4.66% and 40.27 to 43.90%, respectively. Kale exhibited higher carotenoid levels, particularly lutein (996.36 mg/100 g) and beta-carotene (574.15 mg/100 g). In comparison, Sango radish excelled in glucose metabolism (α-glucosidase inhibition: 58%) and antioxidant activities (DPPH^•^: 7.92 mmol TE/100 g, ABTS^•+^: 43.47 mmol TE/100 g). Both showed antimicrobial activity against *Escherichia coli* and *Staphylococcus aureus*. Kale demonstrated stronger anti-inflammatory effects, while Sango radish showed antiproliferative potential. These results, supported by PCA and correlation analysis, underscore the dual role of these microgreens as nutritious and therapeutic food additives, addressing oxidative stress, inflammation, and microbial threats.

## 1. Introduction

The rising interest in functional foods and natural sources of antioxidant and nutritional compounds, driven by consumer demand for health-promoting options, has highlighted microgreens as valuable additions to the market due to their high nutritional content and health benefits. Microgreens are a relatively new type of product and they have gained popularity for their vibrant colors, intense flavors, and ability to enhance the appearance and taste of various dishes. These young seedlings of vegetables, herbs, or grains are highly nutritious, containing significantly higher levels of vitamins, minerals, antioxidants, and phenolic compounds compared to their seeds and mature counterparts [1]. Antioxidant compounds are particularly abundant in microgreens and contribute significantly to their appeal as functional foods. They have been associated with various health benefits such as anti-inflammatory, antioxidant, anticancer, and anti-obesity properties. Therefore, microgreens’ potential is highly attributed to their rich content of these bioactive compounds, which include vitamins C, E, and K, beta-carotene, lutein, phenolic acids, flavonoids, and glucosinolates [2].

Microgreens are harvested shortly after the cotyledon leaves appear, typically within 7–14 days. Moreover, the ability to grow microgreens in controlled environments with minimal resource inputs makes them a sustainable option for urban agriculture and food security initiatives. Additionally, quick and efficient production, coupled with dense nutritional value, provides a solution to the malnutrition driven by the fast-paced lifestyles of the modern world [3]. One of the critical factors in the successful application of microgreens in functional foods is species selection. Different microgreen species exhibit varying levels of nutritional and phytochemical properties, influencing their functional quality and health benefits. Brassicaceae family members stand out as one of the most promising microgreen species, due to their high glucosinolate and phenolic content, connected to the antioxidant and anticancer properties [4,5]. Consumer acceptance is vital, with factors like taste, texture, color, and perceived health benefits playing key roles. Educating consumers on microgreens’ nutritional benefits can enhance their diet integration [6].

Despite growing interest in microgreens, significant gaps remain in understanding their nutritional profiles and biological activities. This study aims to address these gaps by comprehensively evaluating the nutritional and antioxidant profiles of selected Brassicaceae microgreens. The first domain of this research uses fast spectrophotometric methods with standard score evaluations to select appropriate species and assess their suitability for functional food products. By further employing more advanced analytical techniques as well as sophisticated statistical analyses, this research seeks to identify correlations between chemical composition and bioactive potential, contributing to the development of microgreens as sustainable, health-promoting functional foods.

## 2. Materials and Methods

### 2.1. Microgreen Samples

Microgreens were selected based on their nutritional profiles and availability in the Serbian market, sourced from the agricultural producer “Mikro Salaš” in Novi Sad, Serbia. The study included kale (*Brassica oleracea* var. *acephala*), mustard (*Brassica juncea*), Daikon radish (*Raphanus sativus* var. *longipinnatus*), red cabbage (*Brassica oleracea* var. *capitata*), kohlrabi (*Brassica oleracea* var. *gongylodes*), and Sango radish (*Raphanus sativus* cv. Sango), categorized into green (kale, mustard, Daikon radish) and purple (red cabbage, kohlrabi, Sango radish) groups. Seeds were sourced from certified suppliers, and microgreens were grown under controlled conditions (22 ± 2 °C, 16 h light/8 h dark photoperiod, 200 µmol m^−2^ s^−1^ light intensity) in peat substrate (pH 5–6). After 10 days, microgreens were harvested, weighed, lyophilized, homogenized, and milled for further analysis.

### 2.2. Selection of the Bioactive Compound-Rich Microgreens

A standard score analysis was used to identify microgreens with the highest concentration of bioactive compounds, allowing direct comparison across species. Extracts were prepared using a 50:50 ethanol–water solution to measure phenols, flavonoids, anthocyanins, vitamin C, carotenoids, and chlorophylls. Namely, the microwell-adapted spectrophotometer Multiscan GO was used to quickly determine present phytochemicals relevant to standard score analyses selection and screening of microgreens (Thermo Fisher Scientific Inc., Waltham, MA, USA). The total phenolic content from the microgreen samples was established using a microwell plate adaptation of the Folin–Ciocalteu method [7]. The reaction mixture was prepared by combining 15 μL of extract, 170 μL of distilled water, 12 μL of Folin–Ciocalteu reagent (2 M), and 30 μL of 20% sodium carbonate in 96-well microplates. After 1 h, absorbance was recorded at 750 nm, with distilled water as the blank. Results were expressed as gallic acid equivalents (GAEs) per 100 g of dried sample. Markham’s modified method was used to spectrophotometrically determine flavonoids’ presence in microgreen extracts using aluminum chloride and reagent, as described in detail by the work of Tumbas et al., 2020 [8]. The concentration of flavonoids is determined based on the difference in absorbance between the test sample and the blank, as read from the calibration curve generated using a standard rutin solution. Vitamin C analyses were performed as per the method described in the work of Šeregelj et.al. [9], where a volume of 0.2 mL of the extract was added to 0.8 mL of 10% trichloroacetic acid, following the 5 min centrifugation and addition of 0.2 mL of 0.2 N Folin–Ciocalteu reagent and 1.5 mL of water to 0.5 mL of the supernatant. After 10 min, absorbance was measured at 765 nm and results were expressed using L-ascorbic acid calibration curve. The total anthocyanin content was determined using a single pH method, where the absorbance of the solution at pH 1 is proportional to the total anthocyanin content [10]. A 25 μL sample solution was placed into a microtiter plate well and diluted to 250 μL with pH 1 buffer and, after 15 min, absorbances were measured at 510 nm and 700 nm. Calculations are expressed as cyanidin-3-glucoside equivalents (mg/L of sample). Total carotenoids and chlorophyll content are measured spectrophotometrically by directly measuring prepared extracts in microwell plates. For total carotenoids, absorbances were measured at 663, 645, 515, and 453 nm, and results were expressed as mg of β-carotene per 100 g of sample and calculated based on the following equation [11]:

C _(mg β-carotene/100 mg)_ = 0.216 × A_663nm_ − 1.22 × A _645nm_ − 0.304 × A _505nm_ + 0.452 × A_453_ (1)

Total chlorophylls, as well as chlorophyll a and b, were determined at two wavelengths (663 and 645 nm), and calculated as chlorophylls equivalent based on the following equations [12]:

TChl (mg/100 mL) = 20.20 A_645_ + 8.02 A_663_ (2)

Chl a (mg/100 mL) = 11.24 A_663_ − 2.04 A_645_ (3)

Chl b (mg/100 mL) = 20.13 A_645_ − 4.19 A_663_ (4)

### 2.3. Health-Promoting Potentials

#### 2.3.1. Antioxidative Activity

The antioxidant potential of the microgreens was investigated using DPPH^•^, ABTS^•+^, and reducing power (RP) assays. Results were measured via spectrophotometry at different wavelengths and expressed as mmol Trolox equivalents (TE) per 100 g of dried sample. For DPPH^•^, 250 μL of methanol solution (0.89 mM) was mixed with 10 μL of the sample, with absorbance measured at 515 nm after 50 min. ABTS^•+^ scavenger activity was assessed by adding 250 μL of ABTS^•+^ solution to 2 μL of the sample, with absorbance recorded at 414 nm after 35 min. Reducing power was determined by incubating 75 μL of sample with sodium phosphate buffer and potassium ferricyanide at 50 °C for 20 min, followed by absorbance measurement at 700 nm [7].

#### 2.3.2. Antiproliferative Activity

Human colon carcinoma cells (HCT116) and non-tumor human fetal lung fibroblast cells (MRC-5) were maintained as a monolayer culture in Roswell Park Memorial Institute (RPMI) 1640 nutrient medium (Sigma Chemicals Co., St. Louis, MO, USA). RPMI 1640 nutrient medium was prepared in sterile deionized water, supplemented with penicillin (192 IU/mL), streptomycin 200 µg/mL), 4-(2-hydroxyethyl) piperazine-1-ethanesulfonic acid (HEPES) (25 mM), l-glutamine (3 mM) and 10% of heat-inactivated fetal calf serum (FCS) (pH 7.2). The cells were grown at 37 °C in 5% CO_2_ and humidified air atmosphere. The antiproliferative activity of kale and Sango radish extracts was tested using the MTT assay [13]. HCT116 (5000 cells/well) and MRC-5 (3000 cells/well) were seeded in 96-well plates and incubated for 24 h. Extracts were prepared in DMSO, diluted to concentrations up to 1000 µg/mL (Sango radish) and 600 µg/mL (kale), with a final DMSO concentration not exceeding 1%. After 72 h of incubation, MTT solution was added, followed by 4 h of incubation at 37 °C. Formazan crystals were dissolved using 10% SDS, and absorbance was measured at 570 nm. Results were displayed as cell survival percentages. Morphological changes were observed with an inverted microscope (Olympus CKX53, Tokyo, Japan) equipped with a Olympus EP50 camera.(Olympus, Center Valley, PA, USA) after 72 h of treatment.

#### 2.3.3. Neuroprotective Activity

The neuroprotective capabilities of the extracts were assessed using a modified Ellman’s method to measure AChE inhibition [14]. A mixture of 20 µL AChE (0.5 U/mL), 110 µL Tris-HCl buffer (pH 8), and 10 µL extract (up to 33.33 mg/mL) was incubated at 37 °C for 15 min. A blank used Tris-HCl buffer (pH 7.5) instead of AChE, and the control replaced the extract with Tris-HCl buffer (pH 8). After incubation, 40 µL of 5,5′-dithiobis (2-nitrobenzoic acid) and 20 µL of acetylthiocholine iodide were added, with absorbance measured at 412 nm. Galantamine served as a positive control for IC_50_ comparison.

#### 2.3.4. Antidiabetic Activity

The antidiabetic activity was evaluated by inhibiting α-amylase and α-glucosidase using well-known protocols [15,16]. Briefly, for α-amylase inhibition, 90 µL of α-amylase (0.1 µg/mL) was mixed with 80 µL of starch solution and 10 µL of extract (5 mg/mL) or acarbose (0.125–3 mg/mL). After incubation at 37 °C for 10 min, the reaction was stopped with 100 µL of 1 M HCl, followed by 20 µL of Lugol solution, and absorbance was measured at 620 nm. For α-glucosidase inhibition, 100 µL of phosphate buffer (pH 6.8) was mixed with 10 µL of α-glucosidase (0.1 U/mL), 20 µL of extract (20 µg/mL) or acarbose (0.310–10 mg/mL), and 20 µL of p-nitrophenyl α-D-glucoside. After incubation, 80 µL of Na_2_CO_3_ was added, and absorbance was recorded at 400 nm. Results were expressed as the percentage of inhibition and mg/g ACAE of dry extract. All tests were performed in triplicate.

#### 2.3.5. Antihypertension Activity

Antihypertensive activity was assessed by measuring ACE (Angiotensin-Converting Enzyme) inhibition using a UV spectrophotometer [17]. A total of 50 µL of ACE (25 mU/mL) was incubated with 50 µL of the extract at 37 °C for 10 min, followed by the addition of 150 µL of substrate solution (HHL in sodium borate buffer). After 30 min at 37 °C, the reaction was stopped with 250 µL of 1 M HCl. Ethyl acetate was added, centrifuged, and the upper layer was collected and evaporated. The resulting hippuric acid was dissolved in distilled water, and absorbance was measured at 228 nm. Captopril served as the standard.

#### 2.3.6. Antihypercholesteromic Activity

Antihypercholesteromic activity was determined by measuring the decrease in NADPH absorbance in the presence of HMG-CoA reductase. Test solutions with varying concentrations of the extract were mixed with a reaction mixture containing NADPH, HMG-CoA, and HMGR. Absorbance was measured at 340 nm every 20 s for 10 min at 37 °C to evaluate the rate of enzyme inhibition.

#### 2.3.7. Anti-Inflammatory Activity

The anti-inflammatory properties were assessed using the method of Ullah et al. [7]. A reaction mixture containing 2 mL of sample extract, 2.8 mL of phosphate buffer (pH 6.4), and 0.2 mL of fresh egg albumin was incubated at 37 °C for 15 min, then heated to 70 °C. Absorbance was measured at 660 nm using a Multiscan GO plate reader.

### 2.4. Antimicrobial Properties

#### 2.4.1. Antibacterial Activity

The antibacterial activity of microgreens was tested using the agar well diffusion method [18] against *Escherichia coli*, *Staphylococcus aureus*, *Listeria monocytogenes*, and *Salmonella* Typhimurium. Bacterial suspensions were adjusted to 0.5 McFarland standard and spread onto Mueller–Hinton agar. Sterile cellulose disks were impregnated with 15 µL of test extracts and incubated at 37 °C for 24 h. Inhibition zones were measured in millimeters. Experiments were performed in triplicate, with standard antibiotics as controls.

#### 2.4.2. Antifungal and Anti-Yeast Activities

Antifungal and anti-yeast activities were tested using the same disc diffusion method against *Aspergillus niger*, *Penicillium aurantiogriseum*, *Saccharomyces cerevisiae*, and *Candida albicans*. Fungal spores and yeast were cultured and applied to Sabouraud Dextrose Agar plates. Discs with 15 µL of test compounds were incubated at 25 °C for 72 h, with inhibition zones measured in millimeters. Actidione served as a positive control.

#### 2.4.3. Antibiofilm Activity

Antibiofilm activity was assessed using a crystal violet staining assay on biofilms formed in 96-well plates. After treatment with test compounds, the biofilms were stained, and optical density at 590 nm was measured to quantify inhibition [19].

#### 2.4.4. Antiadhesion Activity

Antiadhesion activity was evaluated by incubating bacterial cultures in 96-well plates with test compounds. After 2 h, non-adherent bacteria were washed away, and adherent bacteria were stained with crystal violet. The reduction in bacterial adhesion was calculated based on OD measurements at 590 nm [19].

### 2.5. Nutritive Profile of Microgreens

#### 2.5.1. Amino Acids

Amino acid analysis was obtained by using the standardized ISO 13903:2011 procedure [20] on an Industrial Control Sistem (ICS 5000, Thermo Scientific, Waltham, MA, USA) with column AminoPac PA10 guard. The total used sample mass was 50 mg.

#### 2.5.2. Metals and Metalloids

Iron, zinc, magnesium, calcium, potassium, and sodium contents were measured by an atomic absorption spectrophotometer (Varian SPECTRAA–10, Palo Alto, CA, USA) under the manufacturer-recommended parameters. This adhered to 1000 ppm standards from AccuStandard (New Haven, CT, USA) with calibration curves within a linear range (r = 0.999), following the AOAC method [21].

#### 2.5.3. Content of Carbohydrates (Mono- and Disaccharides)

To investigate total carbohydrate content in microgreen samples, the official ISO/11292:1995 [22] method was followed, including the use of HPLC coupled with an anion-exchange column and pulsed amperometric detection.

#### 2.5.4. Proteins

The protein content of selected microgreens was uncovered using the Kieladl method described in the work of Marcó et al. [23], and by the Kjeldahl Nitrogen Analyzer (BKN-983, BIOBASE, Karnataka, India). The conversion of nitrogen to protein content included a conversion factor of 6.25.

#### 2.5.5. Headspace Analysis of Biogenic Volatile Organic Compounds

Semi-quantitative screening of biogenic volatile organic compounds (BVOCs) is analyzed using a GC/MS technique using a headspace and SPME data preparation and injection techniques. Two grams of lyophilized and powdered plant material was put in the glass tube, crimped, and heated for 30 min at 60 °C with Solid-Phase Micro Extraction (SPME) fiber to absorb volatile compounds from the air. After half an hour, SPME fiber is directly injected in GC/MS, and held for two minutes in the inlet at 290 °C. The SPME fiber is conditioned/cleaned, even though the blanks were not clean, without organic compounds. Likewise, the same procedure was used between species to clean the fiber. BVOCs were quantified by an Agilent gas chromatograph (GC) 5975C coupled with a mass spectrometer (MS) 7890 system (Agilent Technologies Inc., Santa Clara, CA, USA) in spitless mode. All samples were analyzed in SCAN mode. A 30 m long capillary HP-5MS column (0.25 mm × 0.25 μm; Hewlett-Packrad, Palo Alto, CA, USA) was used with high-purity helium as the carrier gas at constant flow at 1 mL min^−1^. A mass spectrometer was used in electron ionization (EI) mode at 70 eV. The transfer line was kept at 280 °C. The initial column temperature was held at 40 °C for 1 min. The temperature ramp was 20 °C min^−1^ to 300 °C. Finally, the temperature was held at 300 °C for 5 min. The whole run lasted 19 min.

### 2.6. Phytochemical Profile

The bioactive compounds in the two selected microgreen samples were analyzed using Shimadzu Prominence HPLC with a DAD detector. Extracts were filtered through a 0.45 μm membrane, and all analyses were run in triplicate. Polyphenols were separated on a Luna C-18 RP column, and chromatograms were recorded at 280 nm (hydroxybenzoic acids) and 320 nm (hydroxycinnamic acids). The mobile phase consisted of acetonitrile and 1% formic acid, with a gradient flow rate of 1 mL/min. Vitamin C and dehydroascorbic acid were separated on a NUCLEODUR Shinx RP column, with a flow rate of 0.4 mL/min at 37 °C. Chromatograms were recorded at 265 nm, and results were expressed as milligrams of ascorbic acid per gram of dried sample. Carotenoids were determined using a Luna C-18 RP column, with a flow rate of 1.5 mL/min at 36 °C, and chromatograms recorded at 473 nm [24,25].

### 2.7. Statistical Analysis

Data analysis was conducted using the STATISTICA 10.0 software (StatSoft Inc., Tulsa, OK, USA), with all measurements taken in triplicate and results shown as mean values with standard deviations (SDs). Variance homogeneity was tested with Levene’s test, and normal distribution with the Shapiro–Wilk test. Analysis of variance (ANOVA) and Tukey’s HSD post hoc test were used to evaluate differences among microgreen samples, identifying statistically significant variations. Principal Component Analysis (PCA) was also conducted to highlight differences and groupings, particularly among kale and Sango radish samples [26,27,28]. For visualizing relationships among health-promoting potentials, antimicrobial properties, and chemical and phytochemical contents, a color correlation analysis was performed in R software version 4.0.3 (64-bit). Microgreens were ranked by standard scores (SSs), with the top-ranked green and purple samples undergoing further analysis for biological activity and chemical profiles based on SS analysis.

## 3. Results and Discussion

### 3.1. Selection of Microgreen Samples Rich in Bioactive Compounds

In the initial stage of this study, bioactive-rich microgreens were selected to represent each group. Specifically, three green microgreens (black mustard, kale, and Daikon radish) and three purple ones (kohlrabi, Sango radish, and red cabbage) were assessed for bioactive compounds, including phenols, flavonoids, anthocyanins, vitamin C, carotenoids, total chlorophyll, chlorophyll a, and chlorophyll b. Known for their antioxidant and antimicrobial properties, phenols, flavonoids, and anthocyanins significantly enhance these plants’ health benefits, which is why understanding each plant’s phytochemical composition is important [29,30]. Vitamin C, a key antioxidant, and carotenoids, essential pigments, were also measured. Chlorophyll levels, such as total chlorophyll, chlorophyll a, and chlorophyll b, were analyzed to gauge green pigment content in these microgreens.

**Figure 1:**
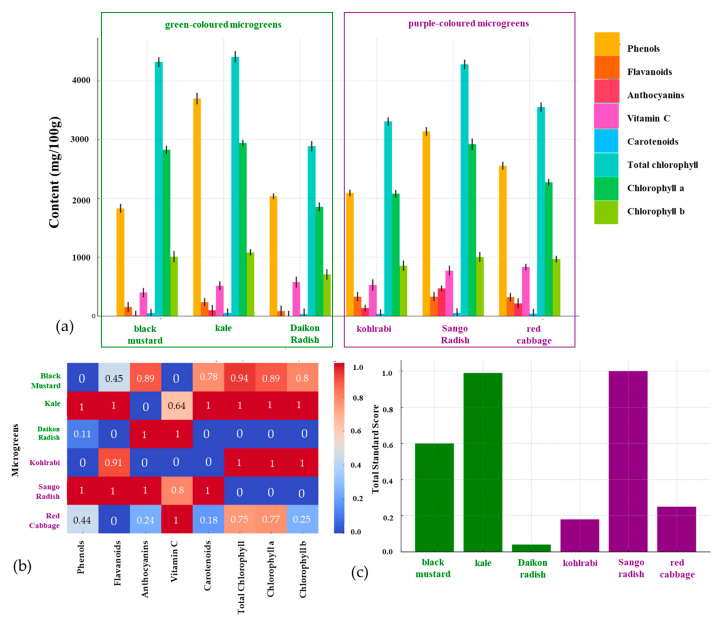
Selection of the best green- and purple-colored microgreen samples based on (**a**) bioactive contents; (**b**) individual standard scores; (**c**) total standard scores.

Experimentally obtained values and standard score evaluations are shown in Figure 1, with datasets available in Supplementary Tables S1 and S2. The analysis of green microgreens—black mustard, kale, and Daikon radish—focused on bioactive compounds. Black mustard had high chlorophyll and carotenoid levels, with moderate phenols, flavonoids, and vitamin C. Kale showed the highest levels of phenols, flavonoids, vitamin C, carotenoids, and chlorophyll, while Daikon radish had high vitamin C but lower carotenoid and chlorophyll. In purple microgreens, kohlrabi had high chlorophyll content with moderate phenols, flavonoids, anthocyanins, and vitamin C. Sango radish led in phenols, flavonoids, anthocyanins, vitamin C, and carotenoids, but had moderate chlorophyll levels. Red cabbage exhibited a balanced profile with high phenols, flavonoids, vitamin C, and chlorophyll. For standard score evaluation, polarity indicated the bioactive effect direction, while coefficients assigned weights in the total score. Kohlrabi scored high in chlorophyll but lower in other compounds. Sango radish excelled in all but chlorophyll, achieving the highest score (1.00), followed by red cabbage (0.25) and kohlrabi (0.18). Among green microgreens, kale ranked highest with a score of 0.99, with black mustard and Daikon radish scoring 0.60 and 0.04, respectively.

The highest-ranked microgreens from both tested groups were selected for further analysis based on comprehensive bioactive profiling visualized in Figure 1, which highlights compound distribution across microgreens (Figure 1a), correlations between bioactives (Figure 1b), and overall ranking by standard score (Figure 1c). This selection captures diverse bioactive profiles within each color group, facilitating a nuanced understanding of their health benefits and applications. Among green microgreens, kale stands out with the highest levels of phenols, flavonoids, vitamin C, carotenoids, and chlorophyll, suggesting potent antioxidant properties and health benefits such as reduced oxidative stress, immune support, eye health, and detoxification. In purple microgreens, Sango radish emerged as the top candidate, rich in phenols, flavonoids, anthocyanins, vitamin C, carotenoids, and moderate chlorophylls. Its anthocyanin abundance offers strong antioxidant benefits, including reduced cardiovascular and cancer risks. Kale’s nutrient density and antioxidant capacity align with studies in the literature highlighting its high vitamin C, lutein, beta-carotene, and phenolic content [31]. Similarly, Sango radish’s abundance of anthocyanins and immune benefits are consistent with studies on purple radish varieties and their role in reducing oxidative stress and inflammation [32,33].

### 3.2. Comprehensive Assessment of Sango Radish and Kale Potentials

#### 3.2.1. Evaluation of Health-Promoting and Antimicrobial Potentials

For an evaluation of health-promoting potentials, the following parameters were included: antioxidant activities (DPPH^•^, ABTS^•+^, RP) and anti-inflammatory activity, as well as activities related to neuroprotective activity, antidiabetic activity (α-amylase and α-glucosidase inhibition), antihypertension and antihypercholesteromic activities, as shown in Table 1. Also, the antimicrobial potential was investigated using anti-yeast, antibacterial, antifungal, antibiofilm, and antiadhesion activities (Table 1).

Sango radish samples show higher antioxidant activity than kale, as indicated by the higher DPPH^•^ and ABTS^•+^ values. For example, Sango radish has the highest ABTS^•+^ activity (43.47 ± 1.26 mM TE/100 g), significantly outperforming Kale samples, which exhibit ABTS^•+^ values around 20 mM TE/100 g. These results suggest that Sango radish might be more effective in scavenging free radicals, which aligns with the known potent antioxidant properties of cruciferous vegetables like radish, as reported in previous studies [34]. On the other hand, kale samples demonstrate much higher reducing power (around 20 mM TE/100 g) compared to Sango radish (around 7 mM TE/100 g). Reducing power is another measure of antioxidant potential, and these results indicate that kale might be better at donating electrons to neutralize reactive species, which could contribute to its health-promoting effects. This finding is consistent with studies in the literature that emphasize the strong reducing power of kale due to its rich content of bioactive compounds such as flavonoids and phenolic acids [35]. The gained values for anti-inflammatory activity, representing the concentration required to inhibit 50% of the free radicals, are lower in Sango radish (ranging from 75.42 to 79.17 μg/mL) than in kale (ranging from 98.22 to 107.95 μg/mL), indicating stronger antioxidant activity in Sango radish. This suggests that Sango radish is more potent at lower concentrations, supporting its superior antioxidant capabilities as discussed in the previous section. Given enzyme inhibition activities, Sango radish samples show relatively lower α-amylase inhibition but higher α-glucosidase inhibition compared to Kale. This could imply that Sango radish may be more effective in inhibiting the enzymes responsible for postprandial hyperglycemia (α-glucosidase), rather than those involved in starch breakdown (α-amylase). The inhibition of these enzymes is crucial for managing blood glucose levels, and Sango radish’s strong α-glucosidase inhibition aligns with studies indicating the potential of radish extracts in diabetes management [36]. Furthermore, kale exhibits significantly lower antihypertension and antihypercholesteromic activities compared to Sango radish. Lower ACE inhibition suggests that kale may be less effective in managing hypertension compared to Sango radish. On the other hand, Sango radish’s higher antihypercholesteromic activity indicates its potential role in cholesterol management. These findings are consistent with reports emphasizing the hypocholesterolemic effects of radish extracts, owing to their glucosinolate content, which can inhibit enzyme response to cholesterol levels in human blood [37].

The antioxidant activities of both Sango radish and kale are well documented in the literature, with cruciferous vegetables like these often highlighted for their high levels of vitamins C and E, phenolics, and flavonoids, all of which contribute to their health benefits [38]. The stronger α-glucosidase inhibition observed in Sango radish compared to kale also corroborates studies that suggest the efficacy of radish in glucose metabolism regulation, which could be advantageous for individuals with diabetes [36]. Furthermore, the enzyme inhibition activities, particularly the higher HMGCR inhibition by Sango radish, suggest that this microgreen could be beneficial in managing cholesterol levels, potentially reducing the risk of cardiovascular diseases. These results align with previous research that identifies radish as a potential functional food for hyperlipidemia and hypertension management [37]. 

Antimicrobial testing using inhibition zone evaluation and antibiofilm and antiadhesion activities (Table 1) is conducted for the assessment potential of kale and Sango radish microgreens for the inhibition of food-borne and spoilage microorganisms related to food (bacteria: *Escherichia coli*, *Staphylococcus aureus*, *Listeria monocytogenes* and *Salmonella* Typhimurium; yeasts: *Saccharomyces cerevisiae* and *Candida albicans*; and fungi: *Aspergillus niger* and *Penicillium aurantiogriseum*). Because of inhibition potential, a significant positive response is observed for *E. coli* and *S. aureus*, and a moderate response for S. Typhimurium, while other tested microorganisms were resistant against tested microgreens samples. A substantial difference is defined for *E. coli* inhibition effect, where Sango radish microgreens have double-size zones compared with kale microgreens.

**Figure 2:**
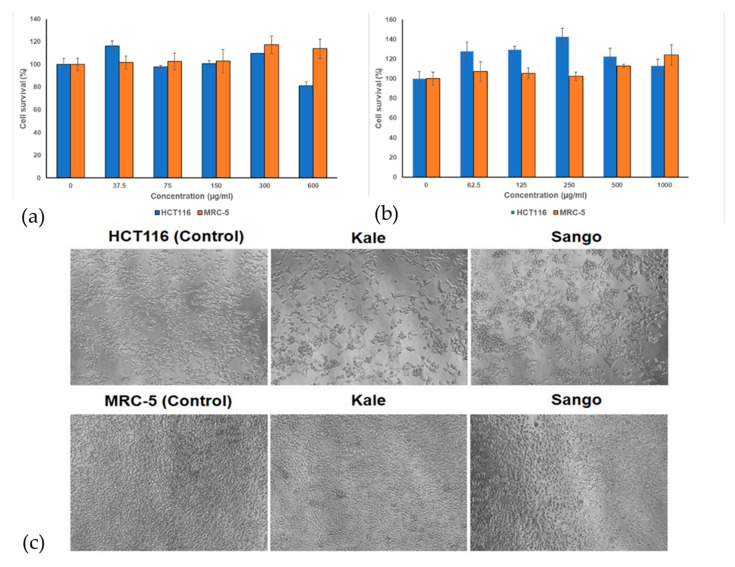
Representative bar graphs of cell survival after 72 h of continual action of (**a**) kale extract and (**b**) Sango radish extract on HCT116 (human colon carcinoma cells) and MRC-5 (non-tumor cells). A representative experiment is shown out of four separate experiments. (**c**) Inverted microscopy examination of HCT116 and MRC-5 exposed to extracts of kale or Sango radish after 72 h using 10×/0.25 objective.

Additionally, the antiproliferative activity of investigated kale and Sango radish extracts was determined in HCT116, human colon carcinoma cells, and MRC-5, a human non-tumor cell line, by MTT assay. The cells were continually incubated for 72 h with extracts. The results of this assay are presented in Figure 2.

Results show that Sango radish extract after 72 h of incubation had no antiproliferative activity on HCT116 and MRC-5 cells in the entire investigated range of concentrations. The extract of kale reduced the survival of HCT116 cells to 80.94% with the highest concentration investigated (600 µg/mL). This activity is in the activity level of DMSO alone, which is used as a solvent for extracts, reducing the survival of HCT116 cells to 80.88% in the final concentration used. The kale extract had no antiproliferative activity on MRC-5 cells in the investigated range of concentrations. The morphological analysis of the HCT116 and MRC-5 cells is presented in Figure 2c. The figure shows that investigated extracts did not affect the number or morphology of non-tumor cells MRC-5. In accordance with the results of the MTT assay, the number of cells was reduced after treatment of HCT116 cells with kale extract, while cells preserved morphology. The investigated extract of Sango radish did not affect the number of HCT116 treated cells, nor their morphology, as per the results of the MTT assay.

#### 3.2.2. Evaluation of Nutritive (Chemical) and Antioxidant Profile

The nutritive profile is evaluated using amino acids, minerals, total proteins, total sugars, glucose, fructose, and other sugars (Table 2). Interestingly, the nutritive profiles of the two microgreens investigated presented many similarities, likely due to their shared classification within the Brassicaceae family. Additionally, the antioxidant profile of the samples was meticulously analyzed using High-Performance Liquid Chromatography (HPLC), focusing on a spectrum of phenolic acids, flavonoids, and carotenoids. Results are presented in Table 3.

Both kale and Sango radish microgreens possess approximate quantities of 17 amino acids, as shown in Table 2. Differences can be noted in the two-times-higher presence of L-isoleucine in Sango radish (7.35%) than in kale (3.58%) and the noticeably higher amount of L-glutamate found in Sango radish (8.47%) compared to kale (5.92%). Comparatively, in the work of Wojdyło et al. [4], from 23 investigated amino acids, L-glutamate was also dominant in kale microgreens (98.8 mg/100 g), while in radish microgreens L-asparagine was the most potent amino acid (221.4 mg/100 g). From the determined mineral composition, the highest amount consisted of potassium, followed closely by calcium. Kale leads over Sango radish in the content of all investigated mineral metalloids except for zinc, with potassium and calcium content as high as 25,634.30 and 19,179.91 mg/kg. The rich mineral composition of Brassicaceae microgreens aligns with the findings of Xiao et al. [39], in which, after examining 30 varieties of microgreens, Brassicaceae family representatives were presented as the highest source of potassium, calcium, iron, and zinc. Following the same trend, total proteins and sugars are found in almost identical amounts in both microgreens, with 40.66–43.83% of total proteins and 4.29–4.66% of total sugars, with glucose and fructose as dominant sugars. Proteins reported in the work of Dereje et al. [40] showed that purple radish and kale microgreens consisted of 3.41 and kale 2.23 mg/100 g fw, respectively. Even though there is an evident lack of studies in the literature on the protein and sugar contents of most microgreens, different Brassicaceae microgreens are found to have variable amounts of glucose, fructose, and sucrose, from 0.5 to 25 mg/g, highlighting the variability of and limited studies in the literature on microgreens’ protein and sugar contents [41].

The evaluation of antioxidant compounds using HPLC revealed distinct differences between Sango radish and kale in the concentrations of various bioactive compounds. In brief, Sango radish showed a significantly high concentration of *p*-hydroxybenzoic acid (510.7 mg/100 g) compared to kale, where this compound was not detected. Similarly, syringic acid and chlorogenic acid were present in Sango radish (29.77 mg/100 g and 80.25 mg/100 g, respectively) but were undetectable in kale. On the other hand, kale was notably richer in catechin (369.16 mg/100 g) and protocatechin (25.07 mg/100 g), both of which were absent in Sango radish. Both plants contained similar levels of gallic acid, caffeic acid, and DHAA, indicating some overlap in their antioxidant profiles. However, kale was particularly high in sinapinic acid (600.88 mg/100 g) and cinnamic acid (96.5 mg/100 g), compared to Sango radish at (333.21 mg/100 g) and (22.95 mg/100 g), respectively. Plenty of researchers have already established the vast potential of Brassicaceae microgreens due to their abundant phenolic content [42,43], including the study of Sun et al. [44], who successfully identified 164 polyphenols (105 flavonol glycosides and 29 derivatives of phenolic acids).

In terms of carotenoids, kale exhibited significantly higher levels of lutein (985.46 mg/100 g), β-carotene (599.99 mg/100 g), and α-carotene (1238.68 mg/100 g), surpassing the concentrations found in Sango radish (511.23 mg/100 g, 90.85 mg/100 g, and 346.15 mg/100 g, respectively). Additionally, kale contained zeaxanthin (125.5 mg/100 g), which was not detected in Sango radish. Furthermore, the presence of alpha and beta carotenoids, and lutein as the main isolated carotenoid, has been confirmed in the literature [4], where the total amounts of alpha and beta carotene were identified as 2239.0 µg/g and 629.6 µg/g, and 699.9 µg/g and 565.2 µg/g of lutein, in kale and radish, respectively. Other identified carotenoids included zeaxanthin, neoxanthin, neochrome, and violaxanthin. Differences in quantity could be explained by geographical varieties as well as differences between fresh and dried plant material. L-ascorbic acid and D-hydroascorbic acid play an important part in microgreens as strong antioxidants. Kale microgreens were recognized as a significantly stronger source of ascorbic acid (66.06 mg/100 g) compared to Sango radish (21.31 mg/100 g), which is supported by multiple studies with kale’s ascorbic acid content varying from 70 to 93 mg/100 fw and radish content going from 15 to 39 mg/100 g fw [45,46]. In a different study where five Brassicaceae microgreens were investigated, total ascorbic acid ranged from 29.67 to 606.87 g AsA_TOT_/g FW [47]. Additionally, variations in ascorbic acid and carotenoid content may depend on post-harvest light exposure. The study of Mlinarić et al. [48] showed a clear example of this, where three types of radish microgreens were examined, demonstrating that light exposure can enhance ascorbic acid levels while reducing carotenoid content.

According to the results gained, Sango radish and kale microgreens are rich in phenolic acids and carotenoids; they differ significantly in the specific types and quantities of these compounds. The differences in phytochemical content could have implications for their respective health benefits and nutritional applications. For instance, the high levels of carotenoids and L-ascorbic acid in kale may enhance its antioxidant capacity, while the unique phenolic acid profile of Sango radish could offer distinct therapeutic properties.

**Figure 3:**
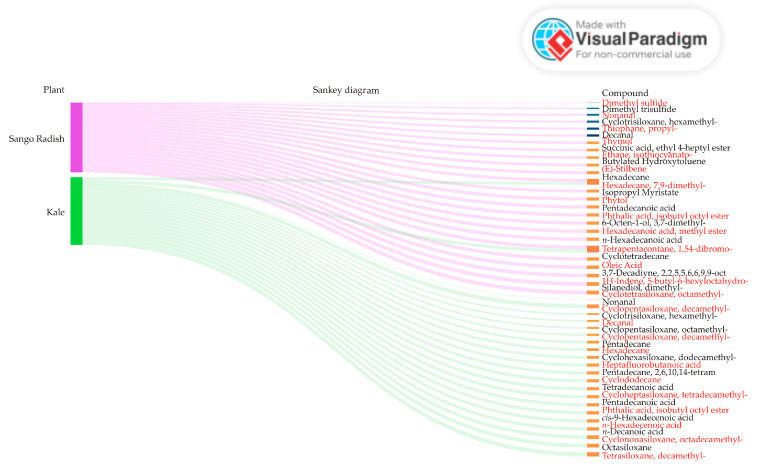
Sankey diagram of aroma-related compounds in Sango radish and kale microgreens obtained by Headspace analysis.

Additionally, the qualitative aroma profiles of Sango radish and kale microgreens reveal significant differences driven by their distinct chemical compositions (Figure 3). The presence of sulfur-containing compounds such as dimethyl sulfide and dimethyl trisulfide in Sango radish microgreens is notable. These compounds are known for their strong, pungent odors, which are often associated with vegetables like onions, garlic, and radishes. Their presence likely contributes to the spicy, sometimes intense, aroma characteristic of Sango radish microgreens. In contrast, kale microgreens exhibit a profile dominated by various siloxanes, including cyclotetrasiloxane, cyclopentasiloxane, and cyclohexasiloxane. These siloxanes are commonly found in personal care products and are known for their neutral, synthetic scents, which may result in a milder and cleaner aroma in kale microgreens. Both Sango radish and kale microgreens share aldehydes such as nonanal and decanal. These compounds are recognized for their citrus-like, fresh odors, which can provide a fresh, fruity base note that balances the overall aroma profile.

Additionally, both microgreens contain fatty acids and their esters, such as hexadecanoic acid, contributing mild, waxy, or fatty scents. These compounds are less intense and likely play a more subtle role in the overall aroma profile of both microgreens. When comparing these findings with those in the existing literature, sulfur compounds have been well documented for their role in the aroma profiles of vegetables, particularly those in the Brassicaceae family, to which radishes belong. The strong, pungent odors they produce are integral to the characteristic aromas of these vegetables [49]. On the other hand, siloxanes, although less commonly discussed in the context of food science, are recognized for their neutral, synthetic odors, often utilized in non-food products [50]. The aldehydes and fatty acids found in both microgreens are widely recognized in the literature for their contributions to fresh, fruity, and fatty aromas in various food products [51]. It can be concluded that the distinct aromatic differences between Sango radish and kale microgreens are dictated by their unique sets of volatile compounds. The sulfur-rich profile of Sango radish microgreens provides a more pungent and intense aroma, while the siloxane-dominated profile of Kale microgreens results in a milder, cleaner scent, underscoring the diversity in aroma profiles even within microgreens.

### 3.3. Comparative Analysis

**Figure 4:**
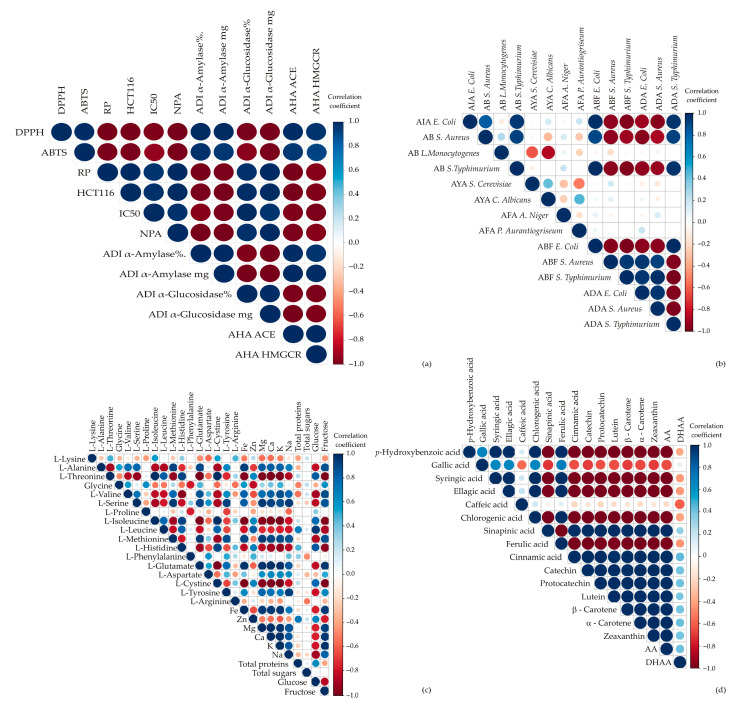
Correlation analysis: (**a**) health-promoting potentials; (**b**) antimicrobial potentials; (**c**) nutritive (chemical) contents; (**d**) antioxidant contents.

The correlation analysis presented in Figure 4 offers a detailed exploration of the health-promoting potentials, antimicrobial activities, chemical contents, and antioxidant profiles of the studied samples. The raw dataset for all tested parameters is given in Table 1, Table 2 and Table 3. The comparison between Sango radish and kale reveals that while both offer health benefits (Figure 4a), Sango radish shows stronger antioxidant activities and enzyme inhibition, potentially enhancing its effectiveness in managing oxidative stress, blood glucose, and cholesterol. Positive correlations between antioxidant activities (DPPH^•^, ABTS^•+^) and α-glucosidase inhibition suggest that these microgreens may aid in blood sugar regulation, consistent with research on polyphenols’ roles in antioxidative defense and glucose management [52].

Interestingly, an inverse relationship between α-amylase and α-glucosidase activities suggests selective inhibition, providing insights for therapeutic development. Neuroprotective activity correlates positively with α-glucosidase inhibition but negatively with α-amylase and ACE inhibition, while antidiabetic activity (α-amylase inhibition) shows positive correlations with antihypertension and antihypercholesteromic activities but negative correlation with α-glucosidase inhibition. Both α-glucosidase and HMGCR inhibition are negatively correlated with ACE inhibition, highlighting distinct therapeutic pathways. Regarding antimicrobial activity (Figure 4b), both microgreens exhibit broad-spectrum effects against *E. coli*, *S. aureus*, and *S*. Typhimurium, with positive correlations among these activities, indicating potential for antimicrobial applications in food products. This is supported by previous findings on the broad-spectrum effects of phenolic-rich microgreen extracts [53,54]. Additionally, *E. coli* inhibition correlates positively with antibiofilm and antiadhesion activities, while *S. aureus* antibiofilm activity correlates positively with overall bacterial antiadhesion activity.

Nutrient profiles (Figure 4c) show strong positive correlations among amino acids (L-lysine, L-glutamate, L-arginine) and between minerals (Fe, Zn, Ca) and protein, suggesting these microgreens are nutritionally balanced sources of essential nutrients. The phytochemical analysis shows co-occurrence among phenolic compounds like gallic and ellagic acids, with a trade-off between *p*-hydroxybenzoic acid and carotenoids such as lutein, likely due to competitive biosynthetic regulation. Similar findings have been observed, indicating potential metabolic prioritization in response to environmental or cultivation factors [55]. Phytochemical content analysis (Figure 4d) highlights positive correlations among phenolic compounds such as gallic, caffeic, and ellagic acids, indicating their co-presence in samples. A negative correlation between *p*-hydroxybenzoic acid and carotenoids (lutein, zeaxanthin) suggests a biosynthetic trade-off, reflecting similar findings in studies on competitive regulation in plant metabolic pathways.

**Figure 5:**
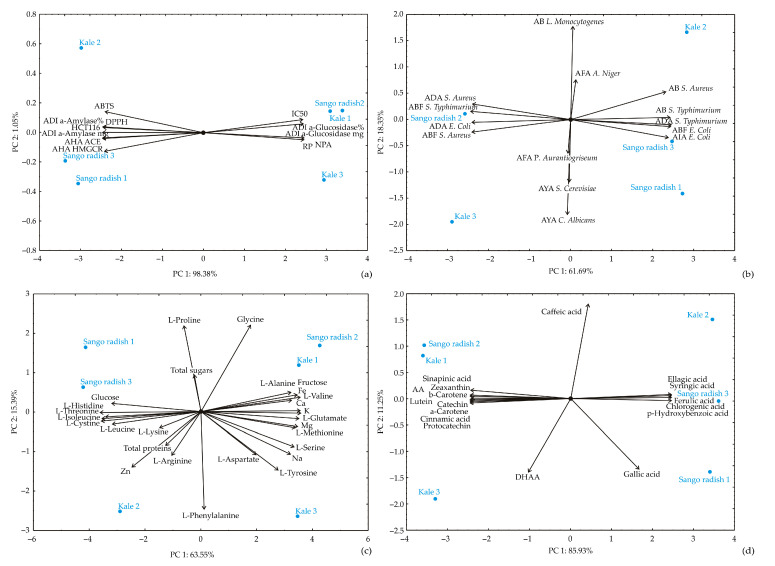
PCA analysis: (**a**) health-promoting potentials; (**b**) antimicrobial potentials; (**c**) chemical contents; (**d**) antioxidant contents.

To further extend the correlation between the obtained results, PCA analysis was conducted (Figure 5). In Figure 5a, PC1 reflects a negative contribution from DPPH^•^, ABTS^•+^, α-amylase inhibition, and ACE inhibition, indicating that higher values for these factors lower PC1 scores. On the positive side, antioxidant activity (reducing power), anti-inflammatory, neuroprotective, and α-glucosidase inhibition contribute positively to PC1. In PC2, ABTS^•+^ activity exerts a strong positive influence, while antihypercholesterolemic activity contributes negatively, with a moderate positive influence from anti-inflammatory activity.

In Figure 5b, PC1 is positively influenced by antibacterial activities against E. coli (10.87%), *S. aureus* (10.34%), and *S*. Typhimurium (11.17%), as well as by antibiofilm activities against *E. coli* (11.35%) and *S.* Typhimurium (11.54%). However, PC1 is negatively affected by antibiofilm activities against S. aureus and *S*. Typhimurium, as well as antiadhesion activities for *E. coli* and *S. aureus*. Figure 5c emphasizes the nutritional profile, where amino acids (e.g., L-alanine, L-valine, L-serine, and L-glutamate) and minerals (Fe, Mg, Ca, and K) positively influence PC1, whereas L-threonine, L-isoleucine, and L-histidine negatively impact it. For PC2, glycine and L-proline have strong positive effects, while L-phenylalanine has a significant negative effect. Figure 5d shows that syringic, ellagic, and chlorogenic acids positively influence PC1, while sinapinic acid, cinnamic acid, and carotenoids like lutein and β-carotene contribute negatively. In PC2, caffeic acid has a strong positive influence, while DHAA and gallic acid contribute negatively. The analysis between Sango radish and kale reveals key bioactive distinctions. Sango radish shows higher antioxidant activities (DPPH^•^, ABTS^•+^) and stronger α-glucosidase inhibition, making it more effective for managing oxidative stress, blood glucose, and cholesterol. The correlation between antioxidant activities and α-glucosidase inhibition suggests a dual role of polyphenols in antioxidative defense and glucose metabolism, consistent with prior research [49]. Additionally, the inverse correlation between α-amylase and α-glucosidase inhibition may indicate a selective inhibition pathway relevant to targeted antidiabetic therapies. The PCA also highlights antimicrobial potential, with positive correlations across antibacterial and antibiofilm activities against *E. coli*, *S. aureus*, and *S.* Typhimurium, highlighting the potential of Sango radish and kale as functional ingredients for antimicrobial applications, such as food packaging. Nutritionally, both microgreens provide a balanced profile of amino acids (e.g., L-lysine, L-glutamate, L-arginine) and minerals (Fe, Zn, Ca), making them valuable for macro- and micronutrient intake. The PCA indicates the positive influence of amino acids and minerals like L-glutamate, Fe, Mg, Ca, and K on nutritional value, supporting Sango radish and kale as nutrient-rich food sources.

## 4. Conclusions

This study comprehensively analyzes the bioactive properties, nutritional content, and potential functional applications of Sango radish and kale microgreens. By using advanced analytical techniques, the research successfully identifies key bioactive compounds and highlights the antioxidant and enzyme inhibition capabilities of these microgreens. The findings show that Sango radish, in particular, demonstrates higher antioxidant activities and stronger enzyme inhibition, positioning it as a capable candidate for managing oxidative stress, blood glucose levels, and cholesterol. These properties suggest that Sango radish may have a valuable role in the prevention of metabolic conditions such as diabetes and cardiovascular disease. Additionally, the obtained results indicated a balanced nutritional profile of both Sango radish and kale, with high concentrations of essential amino acids (e.g., L-lysine, L-glutamate) and minerals (e.g., Fe, Zn, K, Ca), which are significant for their potential as nutrient-dense food sources. This supports their inclusion in health-promoting diets, particularly as plant-based sources of protein and micronutrients. Moreover, the demonstrated antimicrobial properties suggest that these microgreens could be used in the development of functional food products or antimicrobial packaging. Although the obtained results are promising, there is plenty of room for further exploration. The study’s conclusions could be strengthened by including a mechanism of biological activities and conducting in vivo studies in animal models or human trials that would validate the therapeutic benefits of these microgreens. Future research might also explore the bioavailability of the identified nutrients in order to understand their absorption and utilization. Finally, exploring the incorporation of microgreens into functional foods, along with data on their stability throughout processing and storage, would provide support for their industrial applications.

## Figures and Tables

**Table 1:** Evaluation of health-promoting and antimicrobial potentials of Sango radish and kale microgreens.

Health-Promoting Parameters						
Plant Material	Sango Radish 1	Sango Radish 2	Sango Radish 3	Kale 1	Kale 2	Kale 3
Antioxidant activities						
Antioxidant activity, DPPH^•^ (mM TE/100 g)	7.59 ± 0.33 ^b^	7.81 ± 0.23 ^b^	7.92 ± 0.15 ^b^	4.72 ± 0.17 ^a^	4.95 ± 0.29 ^a^	5.03 ± 0.11 ^a^
Antioxidant activity, ABTS^•+^ (mM TE/100 g)	37.89 ± 0.70 ^b^	37.81 ± 1.39 ^b^	43.47 ± 1.26 ^c^	20.22 ± 0.35 ^a^	20.59 ± 0.44 ^a^	19.38 ± 1.35 ^a^
Antioxidant activity, RP (mM TE/100 g)	7.73 ± 0.19 ^a^	7.08 ± 0.43 ^a^	7.45 ± 0.33 ^a^	19.70 ± 0.82 ^b^	19.64 ± 1.35 ^b^	20.84 ± 0.82 ^b^
Pharmaceutical activities						
Anti-inflammatory activity (%)	76.00 ± 4.05 ^a^	75.42 ± 5.24 ^a^	79.17 ± 2.18 ^a^	107.95 ± 3.40 ^b^	104.12 ± 3.32 ^b^	98.22 ± 3.00 ^b^
Neuroprotective activity (mg EE/g de)	10.87 ± 0.21 ^a^	10.26 ± 0.17 ^a^	10.37 ± 0.67 ^a^	14.79 ± 0.11 ^b^	14.40 ± 0.20 ^b^	14.61 ± 0.66 ^b^
ADI a-amylase (%)	19.32 ± 0.46 ^b^	20.92 ± 0.31 ^b^	20.06 ± 0.74 ^b^	12.44 ± 0.54 ^a^	13.26 ± 0.53 ^a^	12.70 ± 0.30 a
ADI a-amylase mg (mg ACAE/g de)	166.15 ± 6.70 ^b^	173.01 ± 7.20 ^b^	162.11 ± 1.41 ^b^	103.12 ± 4.20 ^a^	106.39 ± 2.96 ^a^	109.72 ± 5.06 ^a^
ADI a-glucosidase (%)	54.65 ± 3.68 ^a^	55.95 ± 2.54 ^a^	58.00 ± 2.26 ^a^	84.37 ±2.72 ^b^	85.50 ± 2.10 ^b^	80.03 ± 3.75 ^b^
ADI a-glucosidase mg (mg ACAE/g de)	63.18 ± 2.60 ^a^	57.67 ± 1.58 ^a^	61.24 ± 2.37 ^a^	318.41 ± 18.71 ^b^	311.45 ± 6.47 ^b^	328.40 ± 24.22 ^b^
AHA ACE (%)	44.19 ± 1.03 ^c^	45.29 ± 0.99 ^c^	40.22 ± 1.20 ^b^	14.59 ± 0.31 ^a^	14.91 ± 0.94 ^a^	14.41 ± 0.63 ^a^
AHA HMGCR (%)	53.56 ± 3.04 ^c^	52.84 ± 2.62 ^bc^	47.29 ± 1.65 ^b^	31.97 ± 1.49 ^a^	32.54 ± 1.39 ^a^	34.08 ± 1.30 ^a^
**Antimicrobial Parameters**						
**Plant Material**	**Sango Radish 1**	**Sango Radish 2**	**Sango Radish 3**	**Kale 1**	**Kale 2**	**Kale 3**
Inhibition zone						
*AYA E. Coli* (mm)	19.24 ± 0.45 ^c^	18.96 ± 0.76 ^c^	16.79 ± 1.12 ^b^	10.68 ± 0.59 ^a^	11.04 ± 0.33 ^a^	11.28 ± 0.38 ^a^
*AB S. Aureus* (mm)	25.53 ± 0.67 ^b^	25.63 ± 0.53 ^b^	28.34 ± 0.56 ^c^	21.56 ± 1.34 ^a^	21.91 ± 1.41 ^a^	20.52 ± 0.45 ^a^
*AB L. Monocytogenes* (mm)	6.75 ± 0.21 ^a^	7.09 ± 0.22 ^a^	7.16 ± 0.32 ^a^	7.02 ± 0.44 a	7.21 ± 0.26 ^a^	6.77 ± 0.32 ^a^
*AB S.* Typhimurium (mm)	11.72 ± 0.56 ^b^	10.96 ± 0.16 ^b^	11.31 ± 0.32 ^b^	8.97 ± 0.31 ^a^	9.27 ± 0.34 ^a^	8.75 ± 0.51 ^a^
*AYA S. Cerevisiae* (mm)	7.00 ± 0.14 ^a^	6.92 ± 0.52 ^a^	7.08 ± 0.27 ^a^	7.05 ± 0.29 ^a^	6.72 ± 0.30 ^a^	7.23 ± 0.02 ^a^
*AYA C. Albicans* (mm)	7.33 ± 0.30 ^a^	7.10 ± 0.44 ^a^	6.57 ± 0.41 ^a^	7.11 ± 0.32 ^a^	6.65 ± 0.31 ^a^	7.24 ± 0.11 ^a^
*AFA A. Niger* (mm)	7.10 ± 0.22 ^a^	6.87± 0.12 ^a^	7.04 ± 0.12 ^a^	7.05 ± 0.26 ^a^	7.05 ± 0.42 ^a^	6.89 ± 0.31 ^a^
*AFA P.aurantiogriseum* (mm)	7.27 ± 0.34 ^a^	7.30 ± 0.25 ^a^	6.43 ± 0.31 ^a^	6.81 ± 0.32 ^a^	7.23 ± 0.17 ^a^	6.96 ± 0.51 ^a^
Antimicrobial-correlated Effect						
*ABF E. Coli (%)*	31.38 ± 1.18 ^b^	29.45 ± 1.10 ^b^	29.18 ± 1.07 ^b^	20.25 ± 0.18 ^a^	20.29 ± 0.34 ^a^	19.46 ± 0.68 ^a^
*ABF S. Aureus (%)*	40.79 ± 0.85 ^a^	39.33 ± 1.66 ^a^	39.88 ± 2.93 ^a^	48.96 ± 1.88 ^b^	48.94 ± 1.74 ^b^	52.11 ± 1.83 ^b^
*ABF S.* Typhimurium *(%)*	19.6 ± 0.32 ^a^	20.38 ± 1.18 ^a^	19.93 ± 0.94 ^a^	30.76 ± 1.19 ^b^	30.04 ± 0.97 ^b^	29.20 ± 0.83 ^b^
*ADA E. Coli (%)*	25.71 ± 1.46 ^a^	25.95 ± 0.46 ^a^	23.33 ± 0.92 ^a^	33.61 ± 1.98 ^b^	36.03 ± 1.50 ^b^	35.36 ± 1.61 ^b^
*ADA S. Aureus (%)*	25.89 ± 0.99 ^a^	23.89 ± 1.35 ^a^	25.22 ± 0.41 ^a^	40.28 ± 0.86 ^bc^	41.92 ± 1.72 ^c^	37.80 ± 1.47 ^b^
*ADA S*. Typhimurium *(%)*	15.17 ± 0.49 ^b^	14.85 ± 0.89 ^b^	14.99 ± 0.44 ^b^	5.02 ± 0.38 ^a^	4.93 ± 0.09 ^a^	5.05 ± 0.23 ^a^

DPPH^•^—2,2-diphenyl-1-picrylhydrazyl; ABTS^•+^—2,2′-azino-bis-3-ethylbenzothiazoline-6-sulphonicacid; RP—reducing power (RP); ADI—antidiabetic; AHA ACE—antihypertension activity; AHA HMGCR—antihypercholesteromic activity; AYA—anti-yeast activity; AB—antibacterial activity; AFA—antifungal activity; ABF—antibiofilm activity; ADA—antiadhesion activity. Note: different letters in columns indicate that there is a significant difference at *p* ≤ 0.05.

**Table 2:** Evaluation of nutritive (chemical) profile of Sango radish and kale microgreens.

Plant Material	Sango Radish 1	Sango Radish 2	Sango Radish 3	Kale 1	Kale 2	Kale 3
Amino Acids (%)						
L-Lysine	2.00 ± 0.07 ^a^	2.09 ± 0.09 ^a^	2.18 ± 0.13 ^a^	2.10 ± 0.08 ^a^	1.98 ± 0.09 ^a^	1.96 ± 0.14 ^a^
L-Alanine	2.60 ± 0.14 ^a^	2.73 ± 0.04 ^a^	2.68 ± 0.15 ^a^	2.91 ± 0.09 ^a^	2.97 ± 0.14 ^a^	2.82 ± 0.20 ^a^
L-Threonine	2.67 ± 0.07 ^b^	2.67 ± 0.12 ^b^	2.58 ± 0.04 ^b^	1.46 ± 0.09 ^a^	1.44 ± 0.06 ^a^	1.45 ± 0.05 ^a^
Glycine	2.38 ± 0.14 ^abc^	2.27 ± 0.10 ^abc^	2.16 ± 0.01 ^a^	2.50 ± 0.11 ^c^	2.43 ± 0.05 ^bc^	2.24 ± 0.07 ^ab^
L-Valine	3.76 ± 0.17 ^a^	3.76 ± 0.17 ^a^	3.82 ± 0.12 ^a^	4.22 ± 0.13 ^a^	3.95 ± 0.19 ^a^	4.01± 0.22 ^a^
L-Serine	1.39 ± 0.05 ^a^	1.43 ± 0.05 ^ab^	1.56 ± 0.06 ^bc^	1.67 ± 0.03 ^cd^	1.72 ± 0.06 ^d^	1.77± 0.08 ^d^
L-Proline	1.63 ± 0.04 ^a^	1.57 ± 0.05 ^a^	1.48 ± 0.06 ^a^	1.54 ± 0.07 ^a^	1.62 ± 0.07 ^a^	1.49 ± 0.04 ^a^
L-Isoleucine	7.17 ± 0.24 ^b^	7.29 ± 0.22 ^b^	7.59 ± 0.35 ^b^	3.63 ± 0.22 ^a^	3.60 ± 0.07 ^a^	3.50 ± 0.26 ^a^
L-Leucine	4.13 ± 0.16 ^b^	4.13 ± 0.15 ^b^	3.77 ± 0.09 ^b^	3.32 ± 0.21 ^a^	3.33 ± 0.10 ^a^	3.76 ± 0.08 ^b^
L-Methionine	0.35 ± 0.02 ^a^	0.37 ± 0.01 ^a^	0.37 ± 0.02 ^a^	0.39 ± 0.01 ^a^	0.39 ± 0.01 ^a^	0.39 ± 0.03 ^a^
L-Histidine	0.99 ± 0.04 ^bc^	1.05 ± 0.05 ^c^	0.99 ± 0.02 ^bc^	0.83 ± 0.02 ^a^	0.91 ± 0.03 ^ab^	0.88 ± 0.03 ^a^
L-Phenylalanine	1.16 ± 0.07 ^a^	1.15 ± 0.05 ^a^	1.21 ± 0.05 ^a^	1.14 ± 0.09 ^a^	1.15 ± 0.03 ^a^	1.22± 0.03 ^a^
L-Glutamate	5.69 ± 0.17 ^a^	6.07 ± 0.28 ^a^	6.00 ± 0.37 ^a^	8.46 ± 0.37 ^b^	8.41 ± 0.36 ^b^	8.54 ± 0.22 ^b^
L-Aspartate	3.07 ± 0.24 ^ab^	2.81 ± 0.12 ^a^	2.97 ± 0.15 ^ab^	3.05 ± 0.13 ^ab^	2.99 ± 0.06 ^ab^	3.32 ± 0.08 ^b^
L-Cystine	0.15 ± 0.00 ^b^	0.16 ± 0.01 ^bc^	0.17 ± 0.00 ^c^	0.05 ± 0.00 ^a^	0.05 ± 0.00 ^a^	0.05 ± 0.00 ^a^
L-Tyrosine	1.12 ± 0.04 ^a^	1.15 ± 0.04 ^a^	1.18 ± 0.05 ^a^	1.18 ± 0.08 ^a^	1.18 ± 0.05 ^a^	1.21 ± 0.04 ^a^
L-Arginine	1.92 ± 0.02 ^a^	1.96 ± 0.07 ^a^	2.03 ± 0.06 ^a^	1.91 ± 0.05 ^a^	1.99 ± 0.06 ^a^	1.92 ± 0.04 ^a^
Mineral compounds (mg/kg)						
Iron (Fe)	110.96 ± 5.21 ^a^	117.05 ± 3.50 ^a^	108.30 ±7.25 ^a^	139.53 ± 5.63 ^b^	141.58 ± 5.96 ^b^	137.09 ± 5.30 ^b^
Zinc (Zn)	81.02 ± 6.52 ^ab^	86.96 ± 3.13 ^b^	83.74 ± 3.14 ^ab^	75.35 ± 3.56 ^ab^	74.35 ± 5.10 ^a^	83.82 ± 2.86 ^ab^
Magnesium (Mg)	7078.32 ± 181.11 ^a^	7283.80 ± 290.06 ^a^	7375.88 ± 281.07 ^a^	8842.52 ± 196.28 ^b^	9288.03 ± 359.20 ^b^	9595.45 ± 412.55 ^b^
Calcium (Ca)	11,552.58 ± 232.53 ^a^	11,467.27 ± 333.85 ^a^	11,492.14 ± 737.65 ^a^	17,559.58 ± 314.71 ^b^	17,671.51 ± 805.62 ^b^	19,179.91 ± 1400.02 ^b^
Potassium (K)	19,787.36 ± 1254.36 ^a^	19,273.36 ± 1484.63 ^a^	19,397.29 ± 1121.84 ^a^	25,445.86 ± 1804.38 ^b^	24,432.85 ± 108.91 ^b^	25,634.30 ± 1643.87 ^b^
Sodium (Na)	2694.62 ± 88.15 ^a^	2707.50 ± 79.83 ^a^	3015.89 ± 109.95 ^b^	3165.34 ± 149.11 ^b^	3075.05 ± 18.24 ^b^	3236.61 ± 65.31 ^b^
Proteins and sugars (%)						
Total proteins	42.59 ± 2.12 ^a^	43.84 ± 2.04 ^a^	41.52 ± 1.82 ^a^	40.27 ± 1.41 ^a^	41.83 ± 0.42 ^a^	43.90 ± 1.25 ^a^
Total sugars	4.37 ± 0.09 ^ab^	4.66 ± 0.18 ^b^	4.29 ± 0.05 ^a^	4.59 ± 0.10 ^ab^	4.29 ± 0.18 ^a^	4.40 ± 0.09 ^ab^
Glucose	3.64 ± 0.06 ^c^	3.55 ± 0.04 ^c^	3.25 ± 0.06 ^b^	2.92 ± 0.06 ^a^	3.09 ± 0.08 ^ab^	3.20 ± 0.14 ^b^
Fructose	0.99 ± 0.03 ^a^	0.92 ± 0.04 ^a^	0.97 ± 0.03 ^a^	1.42 ± 0.07 ^b^	1.38 ± 0.06 ^b^	1.31 ± 0.08 ^b^

Note: different letters in columns indicate that there is a significant difference at *p* ≤ 0.05.

**Table 3:** Evaluation of antioxidant profile of Sango radish and kale microgreens.

Plant Material	Sango Radish 1	Sango Radish 2	Sango Radish 3	Kale 1	Kale 2	Kale 3
Phenolic Compounds (mg/100 g)						
p-hydroxybenzoic	532.58 ± 34.43 ^b^	511.06 ± 13.58 ^b^	488.47 ± 18.78 ^b^	0.00 ± 0.00 ^a^	0.00 ± 0.00 ^a^	0.00 ± 0.00 ^a^
Gallic acid	35.52 ± 1.11 ^c^	34.79 ± 1.33 ^bc^	33.82 ± 0.55 ^a^	31.52 ± 1.96 ^ab^	31.27 ± 1.31 ^a^	34.79 ± 0.98 ^bc^
Syringic acid	29.18 ± 1.22 ^b^	30.54 ± 1.43 ^b^	29.60 ± 1.30 ^b^	0.00 ± 0.00 ^a^	0.00 ± 0.00 ^a^	0.00 ± 0.00 ^a^
Ellagic acid	76.11 ± 3.61 ^b^	76.67 ± 2.10 ^b^	78.16 ± 4.88 ^b^	18.04 ± 1.45 ^a^	17.86 ± 0.88 ^a^	17.36 ± 0.52 ^a^
Caffeic acid	16.76 ± 0.17 ^ab^	16.84 ± 0.65 ^b^	18.24 ± 0.75 ^b^	17.52 ± 0.57 ^b^	17.69 ± 0.63 ^b^	15.67 ± 0.83 ^a^
Chlorogenic acid	81.72 ± 4.23 ^b^	79.37 ± 2.15 ^b^	79.67± 4.23 ^b^	0.00 ± 0.00 ^a^	0.00 ± 0.00 ^a^	0.00 ± 0.00 ^a^
Sinapinic acid	319.50 ± 13.11 ^a^	319.79 ± 0.95 ^a^	360.33 ± 13.99 ^b^	619.82 ± 22.02 ^b^	595.37 ±16.47 ^b^	587.44 ± 20.51 ^b^
Ferulic acid	136.09 ± 3.51 ^b^	145.16 ± 3.59 ^b^	137.57 ± 4.33 ^b^	80.34 ± 0.93 ^a^	77.83 ± 5.11 ^a^	84.83 ± 3.06 ^a^
Cinnamic acid	23.66 ± 1.09 ^a^	23.12 ± 0.90 ^a^	22.07 ± 1.00 ^a^	91.73 ± 2.99 ^b^	99.76 ± 1.19 ^c^	98.01 ± 3.44 ^c^
Catechin	0.00 ± 0.00 ^a^	0.00 ± 0.00 ^a^	0.00 ± 0.00 ^a^	386.61 ±14.35 ^c^	371.94 ± 7.03 ^bc^	348.93 ± 17.32 ^bc^
Protocatechin	0.00 ± 0.00 ^a^	0.00 ± 0.00 ^a^	0.00 ± 0.00 ^a^	25.70 ± 1.64 ^b^	23.94 ± 0.70 ^b^	25.58 ± 1.18 ^b^
Carotenoids (mg/100 g)						
Lutein	503.50 ± 26.90 ^a^	500.59 ± 23.88 ^a^	529.60 ± 25.32 ^a^	1010.00 ± 59.60 ^b^	950.02 ± 31.39 ^b^	996.36 ± 71.30 ^b^
ß-Carotene	91.81 ± 4.39 ^a^	95.39 ± 3.79 ^a^	85.36 ± 3.89 ^a^	624.89 ± 47.56 ^b^	600.93 ± 12.89 ^b^	574.15 ± 26.39 ^b^
α-Carotene	350.57 ± 12.95 ^a^	339.59 ± 12.99 ^a^	348.30 ± 11.92 ^b^	1221.47 ± 55.67 ^b^	1254.60 ± 50.50 ^b^	1239.98 ± 33.14 ^b^
Zeaxanthin	0.00 ± 0.00 ^a^	0.00 ± 0.00 ^a^	0.00 ± 0.00 ^a^	126.95 ± 7.47 ^bc^	131.11 ± 4.47 ^c^	118.45 ± 4.73 ^b^
L-ascorbic acid (mg/100 g)						
AA	20.27 ± 0.58 ^a^	21.82 ± 0.59 ^a^	21.84 ± 1.04 ^a^	65.01 ± 2.01 ^b^	66.07 ± 2.67 ^b^	67.11 ± 1.60 ^b^
DHAA	57.97 ± 2.53 ^a^	55.71 ± 2.54 ^a^	55.28 ± 2.20 ^a^	56.50 ± 1.22 ^a^	57.28 ± 1.76 ^a^	57.70 ± 3.98 ^a^

AA—ascorbic acid, dehydroscorbic acid. Note: different letters in columns indicate that there is a significant difference at *p* ≤ 0.05.

## Data Availability

The original contributions presented in the study are included in the article; further inquiries can be directed to the corresponding authors.

## Supplementary Materials

The following supporting information can be downloaded at https://www.mdpi.com/article/10.3390/antiox14020191/s1, Table S1: Standard score analysis for purple-colored microgreen samples; Table S2: Standard score analysis for green-colored microgreen samples.
