# Supplementary material for: A Comprehensive Antioxidant and Nutritional Profiling of Brassicaceae Microgreens
Source: Antioxidants (Basel). 2025 Feb 7;14(2):191. doi: 10.3390/antiox14020191 (PMC11852083; doi:10.3390/antiox14020191)
Supplement: Supplementary file 1 [file antioxidants-14-00191-s001.zip › antioxidants-3462489-supplementary.pdf]

SUPPLEMENTARY MATERIAL

Table S.1. Standard Score Analysis for purple-colored microgreen samples

**Microgreen sample**	**Content of bioactive compounds**							
	**Phenols**	**Flavonoids**	**Anthocyanins**	**Vitamin C**	**Carotenoids**	**Total chlorophyll**	**Chlorophyll a**	**Chlorophyll b**
Kohlrabi	2094.56	328.88	135.98	528.22	35.8	3308.62	2082.31	856.2
Sango Radish	3140.36	329.42	468.14	772.85	51.65	4280.54	2923.19	1006.16
Red Cabbage	2556.18	323.5	216.97	833.91	38.63	3554.45	2274	969.07
Polarity	+	+	+	+	+	-	-	-
Coefficient (∑=1)	0.2	0.2	0.56	0.02	0.02	0	0	0
Min	2094.56	323.5	135.98	528.22	35.8	3308.62	2082.31	856.2
Max	3140.36	329.42	468.14	833.91	51.65	4280.54	2923.19	1006.16
	Individual Standard Score							
Kohlrabi	0.00	0.91	0.00	0.00	0.00	1.00	1.00	1.00
Sango Radish	1.00	1.00	1.00	0.80	1.00	0.00	0.00	0.00
Red Cabbage	0.44	0.00	0.24	1.00	0.18	0.75	0.77	0.25
	Total Standard Score							
Kohlrabi	0.18							
Sango Radish	**1.00**							
Red Cabbage	0.25							

Table S.2. Standard Score Analysis for green-colored microgreen samples

Microgreen sample	Content of bioactive compounds							
	**Phenols**	**Flavonoids**	**Anthocyanins**	**Vitamin C**	**Carotenoids**	**Total chlorophyll**	**Chlorophyll a**	**Chlorophyll b**
Black Mustard	1832.62	153.01	12.12	399.17	47.7	4321.27	2826.65	1010.16
Kale	3700.82	237.18	97.61	514.44	52.22	4409.04	2942.94	1083.65
Daikon Radish	2037.73	82.83	1.42	578.38	31.31	2888.65	1857.72	707.41
Polarity	+	+	-	+	+	+	+	+
Coefficient (∑=1)	0.2	0.2	0	0.02	0.02	0.18666	0.18666	0.18666
Min	1832.62	82.83	1.42	399.17	31.31	2888.65	1857.72	707.41
Max	3700.82	237.18	97.61	578.38	52.22	4409.04	2942.94	1083.65
	Individual Standard Score							
Black Mustard	0.00	0.45	0.89	0.00	0.78	0.94	0.89	0.80
Kale	1.00	1.00	0.00	0.64	1.00	1.00	1.00	1.00
Daikon Radish	0.11	0.00	1.00	1.00	0.00	0.00	0.00	0.00
	Total Standard Score							
Black Mustard	0.60							
Kale	**0.99**							
Daikon Radish	0.04							
